# Genetically predicted protein concentrations in association with Alzheimer's disease risk: Insights from nonlinear protein modeling

**DOI:** 10.1002/alz70856_099503

**Published:** 2025-12-24

**Authors:** Jingjing Zhu, Ben Dai, Unhee Lim, Keenan A. Walker, Xueqiu Jian, Quan Long, Zhongming Zhao, Chong Wu, Lang Wu, Youping Deng

**Affiliations:** ^1^ University of Hawai'i Manoa, Honolulu, HI, USA; ^2^ The Chinese University of Hong Kong, Hong Kong, China; ^3^ University of Hawaii, Honolulu, HI, USA; ^4^ National Institute of Aging Intramural Research Program, National Institutes of Health, Bethesda, MD, USA; ^5^ The University of Texas Health Science Center at San Antonio, San Antonio, TX, USA; ^6^ University of Calgary, Calgary, AB, Canada; ^7^ School of Biomedical Informatics, The University of Texas Health Science Center at Houston, Houston, TX, USA; ^8^ The University of Texas MD Anderson Cancer Center, Houston, TX, USA; ^9^ University of Hawai'i Cancer Center, Honolulu, HI, USA; ^10^ University of Hawai'i at Mānoa, Honolulu, HI, USA

## Abstract

**Background:**

Alzheimer's disease (AD) poses a significant public health burden. Despite extensive research, current therapeutic options offer limited efficacy in halting disease progression. Understanding the systemic physiological changes influencing AD development is crucial for developing innovative strategies for effective treatment. Leveraging genetic variants as instrumental variables, Mendelian randomization and proteome‐wide association study have identified numerous protein biomarkers associated with AD risk, yet potential nonlinear associations have largely been overlooked.

**Method:**

In this study, we applied a nonlinear modeling approach, combining two‐stage sliced inverse regression (2SIR) with nonlinear transformations via adjusted inverse regression (AIR), to investigate associations between genetically predicted protein concentrations and AD risk by integrating data from the INTERVAL study, which contains both blood proteome and genome data, and the summary statistics of large genome‐wide association study of AD.

**Result:**

We identified 131 proteins associated with AD after stringent Bonferroni correction. Of these, 46 had been previously reported using linear modeling methods, highlighting the complementarity of the currently used nonlinear approach. Notably, many of the identified proteins have established biological relevance to AD, including APOE, ADAM11, LRP1B and TREML2, indicating that these critical AD‐associated proteins could only be identified in instrumental variable analysis by allowing for nonlinear associations.

**Conclusion:**

Our study underscores the importance of accounting for nonlinear relationships in uncovering important gene products associated with AD. Our method could improve the understanding of AD pathogenesis and inform future therapeutic and preventive strategies to reduce AD burden.

